# TGFBI secreted by tumor-associated macrophages promotes glioblastoma stem cell-driven tumor growth via integrin αvβ5-Src-Stat3 signaling

**DOI:** 10.7150/thno.69605

**Published:** 2022-05-16

**Authors:** Peng Peng, Hongtao Zhu, Dan Liu, Zirong Chen, Xiaolin Zhang, Zhongyin Guo, Minhai Dong, Lijun Wan, Po Zhang, Guohao Liu, Suojun Zhang, Fangyong Dong, Feng Hu, Fangling Cheng, Shijun Huang, Dongsheng Guo, Bin Zhang, Xingjiang Yu, Feng Wan

**Affiliations:** 1 Department of Neurosurgery, Tongji Hospital of Tongji Medical College, Huazhong University of Science and Technology, Wuhan, Hubei, China.; 2 Department of Medical Genetics, School of Basic Medicine, Tongji Medical College, Huazhong University of Science and Technology, Wuhan, Hubei, China.; 3 Department of Surgery, Hepatic Surgery Center, Tongji Hospital of Tongji Medical College, Huazhong University of Science and Technology, Wuhan, Hubei, China.; 4 Department of Neurosurgery, Shayang County People's Hospital, Jingmen, Hubei, China.; 5 Department of Physiology, School of Basic Medicine, Tongji Medical College, Huazhong University of Science and Technology, Wuhan, Hubei, China.; 6 Hubei Key Laboratory of Drug Target Research and Pharmacodynamic Evaluation, Huazhong University of Science and Technology, Wuhan, Hubei, China.; 7 The Institute for Brain Research, Collaborative Innovation Center for Brain Science, Huazhong University of Science and Technology, Wuhan, Hubei, China.; 8 Department of Histology and Embryology, School of Basic Medicine, Tongji Medical College, Huazhong University of Science and Technology, Wuhan, Hubei, China.

**Keywords:** TGFBI, tumor-associated macrophages (TAMs), glioblastoma (GBM), glioblastoma stem cells (GSCs), integrin αvβ5

## Abstract

**Rationale:** In the glioblastoma (GBM) microenvironment, tumor-associated macrophages (TAMs) are prominent components and facilitate tumor growth. The exact molecular mechanisms underlying TAMs' function in promoting glioma stem cells (GSCs) maintenance and tumor growth remain largely unknown. We found a candidate molecule, transforming growth factor beta-induced (TGFBI), that was specifically expressed by TAMs and extremely low in GBM and GSC cells, and meanwhile closely related to glioma WHO grades and patient prognosis. The exact mechanism of TGFBI linking TAM functions to GSC-driven tumor growth was explored.

**Methods:** Western blot, quantitative real-time PCR (qRT-PCR), enzyme-linked immunosorbent assay (ELISA), immunofluorescence (IF), immunohistochemistry staining (IHC) and public datasets were used to evaluate TGFBI origin and level in GBM. The response of GSCs to recombinant human TGFBI was assessed *in vitro* and orthotopic xenografts were established to investigate the function and mechanism *in vivo*.

**Results:** M2-like TAMs infiltration was elevated in high-grade gliomas. TGFBI was preferentially secreted by M2-like TAMs and associated with a poor prognosis for patients with GBM. TGFBI promoted the maintenance of GSCs and GBM malignant growth through integrin αvβ5-Src-Stat3 signaling *in vitro* and *in vivo*. Of clinical relevance, TGFBI was enriched in the serum and CSF of GBM patients and significantly decreased after tumor resection.

**Conclusion:** TAM-derived TGFBI promotes GSC-driven tumor growth through integrin αvβ5-Src-Stat3 signaling. High serum or CSF TGFBI may serve as a potential diagnostic and prognostic bio-index for GBMs.

## Introduction

Glioblastoma (GBM) is the most frequent and malignant intracranial neoplasm and remains incurable despite maximal therapy 1, 2. Failure of GBM treatment is partly attributable to a small population of cells expressing stemness characteristics, namely, glioblastoma stem cells (GSCs). GSCs are capable of self-renewal and differentiation, highly resistant to conventional treatments and therefore implicated in tumor progression and recurrence 3-7. GSCs dynamically interplay and communicate with multiple noncancerous stromal cells in the tumor microenvironment, among which tumor-associated macrophages (TAMs) in particular are associated with high tumor grade and unfavorable prognosis 8-12. As one of the main regulatory components in the tumor microenvironment, TAMs comprise as many as 30%-50% of all cells in human GBM 9 and play a critical role in tumor formation and maintenance. Interestingly, TAMs and GSCs are found to be co-enriched in hypoxic and perivascular niches and increased in recurrent GBMs, and functionally have a close relationship in conferring tumorigenesis 13-18. According to the functional phenotypes, TAMs could be arbitrarily classified into tumor-suppressive M1-like and tumor-supportive M2-like. 16, 19-21. TAMs in gliomas are commonly skewed toward M2-like TAMs 17.

The close association between TAMs and GSCs suggests reciprocal molecular crosstalk that is crucial for their pro-tumorigenic function. By producing soluble factors such as IL-10 22, 23, stress inducible protein 1 24, IL1β 25, and CCL8 26, TAMs can activate glioma cell-intrinsic signaling responsible for proliferation, invasion and angiogenesis. However, only a few studies report on the molecular mechanism underlying the pro-tumor functions of TAMs on GSCs 18, 27. Interrupting the molecular link mediating TAMs' effect on GSC maintenance may represent a novel and potent therapeutic target to inhibit GSC-driven tumor growth, especially given the phenotypic plasticity of TAMs and the failure of reducing the number of TAMs in the therapeutic studies based on TAM-depleting strategy 15. We found such a candidate molecule, transforming growth factor beta-induced (TGFBI) that was specifically expressed in M2-like TAMs and extremely low in GBM and GSC cells, and meanwhile closely related to glioma WHO grades and patient prognosis. TGFBI is composed of 683 amino acids, and the secreted form has a predicted molecular weight of 68 kDa. It has been shown to participate in a broad spectrum of cellular processes, including inflammation, cell growth, tumor progression and metastasis 28-31.

In the current work, we elaborated on the molecular and signaling mechanism of TGFBI mediating TAMs pro-tumor effect on GSCs and tried to explore the potential application of TGFBI as the index of TAM functionality and tumor burden in the clinical background.

## Methods

### U937 cells and U937-derived M0, M1 and M2 macrophages

U937 cells were grown in Roswell Park Memorial Institute (RPMI) 1640 medium with 10% fetal bovine serum (FBS) in the humidified environment containing 5% CO2 at 37 °C. U937-induced macrophages were utilized to mimic different phenotype TAMs 32-34. Specifically, U937 cells were induced into M0 macrophages using 5nM PMA (Sigma, Cat# P1585) for 48h firstly. Then the M0 macrophages were stimulated with 20 ng/ml of IFN-γ (Peprotech, Cat# AF300-02) and 100 ng/ml of LPS (Sigma, Cat# L4516) for an additional 48 h or a combination of IL-4 (Peprotech, Cat# AF200-04, 20 ng/ml), IL-10 (Peprotech, Cat# AF200-10, 20 ng/ml) and TGF-β (Peprotech, Cat# AF100-21C, 20 ng/ml) for an additional 72 h to establish M1-like or M2-like macrophages respectively 12, 27. The marker genes of M0 (IBA1), M1 (iNOS, CD86), and M2 (CD163, CD206) macrophages were inspected to verify whether U937 cells were effectively polarized. The detailed information about these chemicals was listed in Table S1.

### GBM tumor specimens

Six human GBM specimens were collected with informed consent. Their associated clinical information was summarized in Table S2. The tissue microarray (TMA) containing 78 representative tissue samples (Table S3) was prepared at Tongji Hospital, Huazhong University of Science and Technology. All procedures were performed following the principles of the Helsinki Declaration and approved by the institutional ethics committees.

### GSCs culture and differentiation

GSCs were dissociated and functionally characterized from freshly resected GBM specimens or patient-derived GBM orthotopic xenografts 4, 35, 36. Specifically, cells were isolated from freshly collected tumor specimens or patient-derived GBM xenografts with the Papain Dissociation System as per manufacturer instructions (Worthington Biochemical, Cat# LK003150). The isolated cells were cultured in Neurobasal medium with B27 supplement (BasalMedia, Cat# S441J7), 10 ng/ml EGF (R&D, Cat# 236-EG), 10 ng/ml FGF (R&D, Cat# 4114-TC), 10% penicillin/streptomycin (BasalMedia, Cat# S110JV), 1mM sodium pyruvate (BasalMedia, Cat# 11360070), and 2mM L-glutamine (BasalMedia, Cat# 35050061). To obtain CD133-positive cells, we used magnetically labeled CD133 microbeads (Miltenyi Biotec, Cat# 130-100-857) to separate GSC by magnetically activated cell sorting assay. These CD133-positive cells were cultured in the stem cell medium as depicted above and tested for expression of SOX2, Olig2, Nestin and absence of GFAP expression. After that, functional assays, including the sphere-forming ability, multipotent differentiation, and glioma-initiation in immunodeficient mice, were carried out to validate the cancer stem cell characteristics of the CD133+ cells. Specifically, 456 GSCs were derived from a primary GBM patient (60-year-old, sex not available); 3691 GSCs were derived from a primary GBM patient (59-year-old, female). GSCs were differentiated by culturing GSCs in the serum-containing medium (DMEM with 10% FBS) without growth factors.

### Orthotopic mouse xenografts

The animal experiments were performed under the Institutional Animal Care and Use Committee guidelines and were approved by the Institutional Animal Care and Use Committee (IACUC) of Huazhong University of Science and Technology. Mice utilized in these researches were 4-week-old NU/NU nude mice purchased from Beijing Vital River Laboratory Animal Technology Co., Ltd. All mice were reared in peculiar pathogen-free cages and monitored daily by the veterinary personnel. Five mice were housed per cage, with ad libitum access to water and food. 2 × 10^4^ GSCs and 2 × 10^4^ M2 macrophages were co-injected into the right cerebral cortex of the mouse at a depth of 3.5 mm. Mice were euthanized when neurological symptoms were observed. All surgical procedures were conducted under anesthesia by intraperitoneal injection of a ketamine and xylazine cocktail. The brain samples were fixed in 4% paraformaldehyde for 24 hours, then dehydrated in 30% sucrose for an extra 48 hours, embedded in OCT at -80 °C overnight, and with cryosection thickness of 10 µm 4.

### Immunofluorescence (IF) and immunohistochemistry staining (IHC)

For the Immunofluorescent staining experiments, the prepared cells and frozen tumor sections were fixed in 4% PFA for thirty minutes, washed three times with PBS, and then permeabilized in PBS containing 0.5% Triton X-100 (Solarbio, Cat# T8200) for twenty minutes. Samples were blocked with 5% albumin from bovine serum with 0.5% Triton X-100 in PBS for sixty minutes at room temperature and then incubated with primary antibodies overnight at 4 °C, followed by the corresponding secondary fluorescently labeled antibodies for one hour at room temperature 4. The nuclei were counterstained with DAPI (Invitrogen). Images were acquired via a laser confocal microscope (OLYMPUS, FV1000) and processed utilizing ImageJ software 1.8.0.

The immunohistochemical staining of tissue sections (4 μm) was performed under standard procedures utilizing polymer detection kits and 3,30-diaminobenzene (DAB) detection (Zhongshan Jinqiao). The stained sections were digitally scanned. The immunohistochemical score (IHC-score) was employed to evaluate the expression of target proteins 37.

### Immunoblotting assay (IB)

Collected cells were lysed in RIPA buffer with the addition of phosphatase and protease inhibitors (Thermo Scientific, Cat# 78442). The protein samples were resolved by SDS-PAGE and transferred onto PVDF membranes. Blots were incubated with primary antibodies overnight at 4 °C, followed by HRP-conjugated species-specific antibodies (Santa-Cruz, 1:5000). All immunoblots were carried out no less than three times.

The following antibodies were used: TGFBI (Proteintech for IB, 1:1000; for IHC, 1:100; for IF, 1:100), CD133 (Affinity for IF, 1:100), SOX2 (Proteintech for IB, 1:1000), SOX2 (Abcam for IF, 1:100), Olig2 (Proteintech for IF, 1:200), Ki67 (Proteintech for IF, 1:200), CD163 (Santa cruz for IF, 1:50; for IHC, 1:50), CD163 (Proteintech for IB, 1:1000; for IF, 1:100), CD206 (Proteintech for IB, 1:1000), IBA1 (Cell Signaling for IF, 1:50), CD86 (Proteintech for IB, 1:1000; for IF, 1:50), iNOS (Proteintech for IB, 1:1000), GFAP (Cell Signaling for IF, 1:200; for IB, 1:1000), ITGAV (Abclone for IB, 1:1000; for IF, 1:50), ITGB5(Cell Signaling for IF, 1:1600; for IB, 1:1000), Stat3 (Cell Signaling for IB, 1:1000), phospho-Stat3 (Tyr705) (Cell Signaling for IF, 1:200; for IB, 1:1000), Src (Cell Signaling for IB, 1:1000), phospho- Src (Tyr527) (Cell Signaling for IB, 1:1000; for IF, 1:100), P53 (Santa cruz for IF, 1:100; for IB, 1:500), CCND1 (Cell Signaling for IB, 1:1000; for IF, 1:50). The detailed source of these antibodies was listed in Table S4.

### Lentiviral vector construction

The human TGFBI, ITGAV-specific shRNA sequences were cloned into the pLKO.1 TRC vectors (Addgene, Cat# SHC002), with the shRNA sequences listed in Table S5. The lentivirus packaging and transduction were performed as described previously 4.

### Cell viability assays

One thousand cells were seeded into each well of 96-well plates with 100 μl culture medium 4. Cell viability was assayed utilizing Cell Titer-Glo (Promega, Cat# G7572) at the indicated time. The experiment was performed in triplicate. All data were conducted in triplicate and normalized to day 0 and presented as mean ± standard deviation.

### Tumorsphere formation assays and *in vitro* limiting dilution assay

For tumorsphere formation, GSCs were implanted into 24-well plates with a density of 1,000 cells per well for four days and tumorsphere numbers were assayed. For *in vitro* limiting dilution assay, increasing numbers of GSCs per well (1, 5, 10, 20, 40) were seeded into a 96-well plate with six replicates for each gradient. The sphere formation efficiency of each gradient was calculated 27.

### 5-Ethynyl-2′-deoxyuridine (EdU) incorporation assay

EdU assays were performed with the Cell-Light EdU Apollo567 *in vitro* Kit (RiboBio, Cat# C10310-1) according to the manufacturer's instructions. Cells were seeded into a 24-well plate with three replicates for each group. After incubation with 50 μM EdU for two hours, the cells were fixed in 4% paraformaldehyde and stained with Apollo Dye Solution. DAPI was used to stain the nucleic acids within the cells. Images were acquired via a laser confocal microscope (OLYMPUS, FV1000), and the number of EdU-positive cells was counted.

### RNA isolation and quantitative real-time PCR (qRT-PCR)

Total RNA was extracted using TRIZOL (Invitrogen), then reversely transcribed to cDNA with HiScript II Q RT SuperMix (Vazyme, R223-01) according to the manufacturer's instructions. PCR amplifications were conducted by using ChamQ SYBR Master Mix (Vazyme, Q311-02/03). The housekeeping genes Actin or GAPDH were used for normalization. The primer pairs for qRT-PCR were listed in Table S6. Data are displayed as means ± SD from three independent experiments.

### Enzyme-linked immunosorbent assay (ELISA)

Human TGFBI ELISA kits were purchased from Abcam (ab155426). Assays were performed following the manufacturer's instructions. All samples were conducted in duplicate, and data are presented as medians or means.

### Data availability

The Cancer Genome Atlas (TCGA) and Chinese Glioma Genome Atlas (CGGA) glioma datasets, including RNA-seq and clinical information, were downloaded from GlioVis (http://gliovis.bioinfo.cnio.es/). GSE37475 dataset was downloaded from Gene Expression Omnibus (GEO) (https://www.ncbi.nlm.nih.gov/geo/).

### Bioinformatics analysis

Single-cell RNA-sequencing (scRNA-seq) data of GSE89567 and GSE84465 were analyzed using publicly available platforms TISCH (http://tisch.comp-genomics.org/home/) and EMBL-EBI (https://www.ebi.ac.uk/gxa/sc/home). Gene set Enrichment Analysis was conducted employing an available online application (http:// software.broadinstitute.org/gsea/index.jsp). The ssGSEA (single-sample Gene set enrichment analysis) was implemented to calculate the M2-like TAMs signature score of TCGA and CGGA glioma patients.

### Statistical analysis

Statistical tests, including Student's t-test, one-way ANOVA, Pearson correlation test, and log-rank test were performed using GraphPad Prism software 7.0 (GraphPad Software, Inc.) or R 3.5.3. Data in the barplot or curve are presented as mean±SD as denoted in each analysis. P < 0.05 was taken to indicate statistical significance unless otherwise stated.

## Results

### M2-like TAMs are elevated in high-grade gliomas

**Figure 1:**
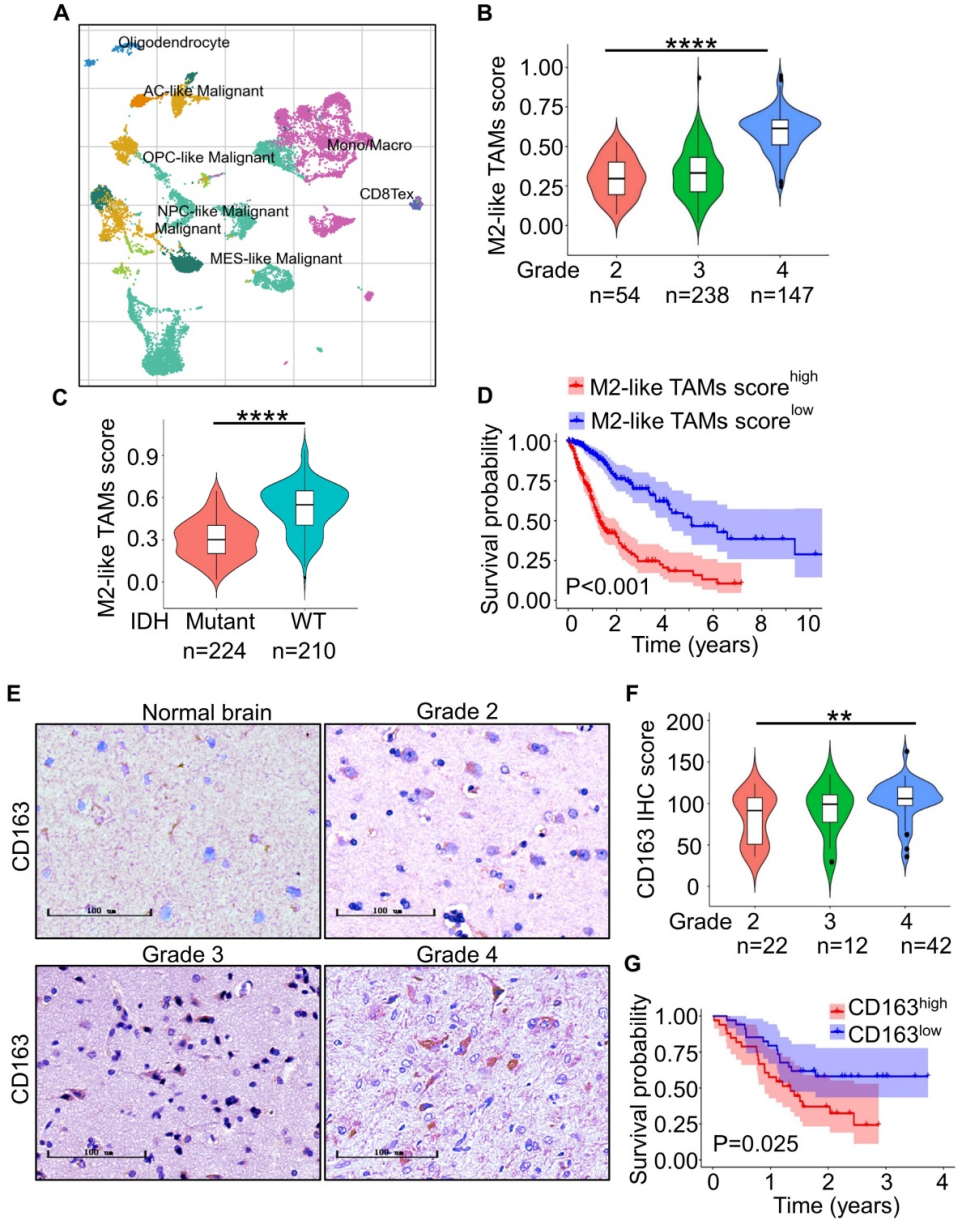
** M2-like TAMs infiltrate more in high-grade gliomas. (A)** t-SNE analysis of 13553 cells of nine glioma patients (GSE131928) showed TAMs accounted for 29.3% of total cells 38. **(B)** ssGSEA of M2-like TAMs signature genes in TCGA-glioma dataset showed M2-like TAMs signature score increased with higher grade gliomas. ****P ˂ 0.0001, ANOVA, analysis of variance. **(C)** Violin plot of M2-like TAMs signature score in IDH-WT and IDH-mutant gliomas from the TCGA database. ****P < 0.0001, Student's t-test. **(D)** Kaplan-Meier survival plots of M2-like TAMs signature score showed a higher score indicated a poorer prognosis. P ˂ 0.001, log-rank test. **(E)** Representative IHC staining of CD163 in glioma microarray. Scale bar represents 20 µm. **(F)** Quantification of the IHC images demonstrated that the score of CD163 increased with increasing WHO grade. **P < 0.01, ANOVA, analysis of variance. **(G)** Kaplan-Meier survival plot of CD163 expression of glioma patients from the glioma microarray. P = 0.025, log-rank test.

To investigate the population of TAMs in gliomas, we first analyzed a single-cell RNA-seq from the GEO database for gliomas (GSE131928) 38 with TISCH. The results showed that TAMs accounted for 29.3% of total cells (Figure 1A). To further elucidate M2-like TAMs infiltration populations, we then evaluated the score of M2-like TAMs signature, which was constructed with eight M2-like TAMs representative genes including CD163, CD206, CD68, CCL18, VEGFA, Fizz1, Arg1, IL10 in gliomas with different grades in TCGA and CGGA glioma cohort with ssGSEA. The result revealed that the M2-like TAMs infiltration was elevated as the tumor grade increased (Figure 1B, Figure S1A) and more M2-like TAMs infiltration in IDH-wildtype (WT) gliomas compared with IDH-mutant gliomas (Figure 1C, Figure S1B). We further investigated the relationship between M2-like TAMs signature and clinic prognosis of gliomas with the Kaplan-Meier analysis. The results demonstrated that M2-like TAMs infiltration was negatively associated with survival for glioma patients (Figure 1D, Figure S1C).

To confirm the above results at the protein level, we performed IHC staining of CD163 in 76 glioma specimens from TMA. The result showed CD163 expression was higher in high-grade gliomas (Grade 3 and 4) in comparison to low-grade gliomas (Grade 2) (Figure 1D and 1E). Importantly, higher CD163 expression informed poorer overall survival in glioma patients (Figure 1F). Together, these data suggest that M2-like TAMs infiltration is increased in high-grade gliomas.

### TGFBI is preferentially secreted by M2-like TAMs and associated with poor prognosis for patients with GBM

**Figure 2:**
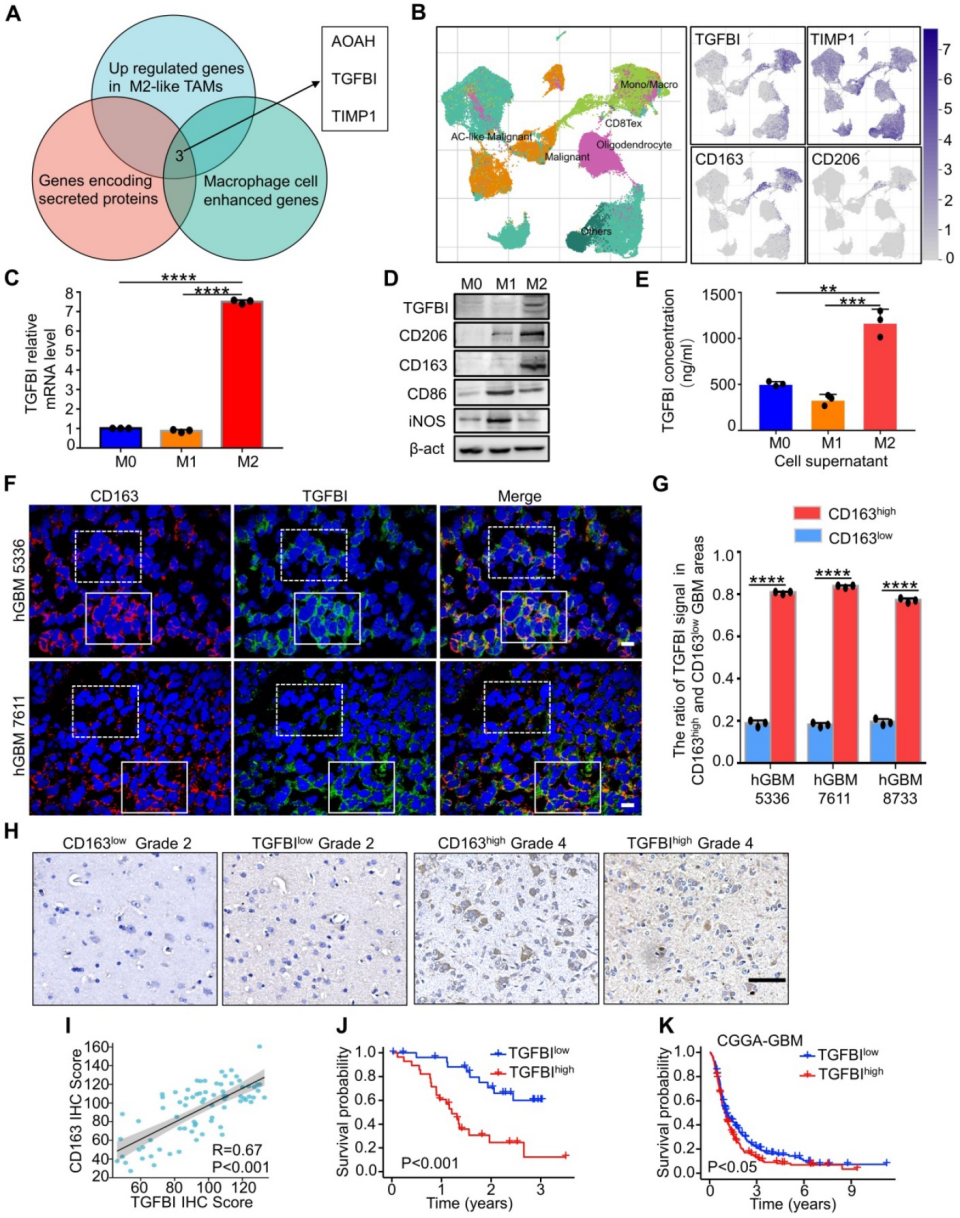
**TGFBI is preferentially secreted by M2-like TAMs and indicates poor prognosis in GBM patients. (A)** Venn diagram demonstrating the overlapping of three datasets, GSE37475 (207 genes upregulated in M2-like TAMs) 15, macrophage cell enhanced genes and genes encoding for secreted proteins from the Human Protein Atlas (http://www.proteinatlas.org), comprised three genes: AOAH, TGFBI, TIMP1. **(B)** t-SNE analysis of 111397 cells of seven glioma patients (GSE148842) 72. Differential coloring in cell clusters was annotated according to the dominant cell type (left). Expression of cell-type-specific TGFBI, TIMP1, CD163 and CD206 overlaid on the t-SNE space (right). **(C)** qRT-PCR analyses of TGFBI expression in M0, M1 and M2 macrophages. N = 3, ****P < 0.0001, Student's t-test. **(D)** Immunoblot analysis of TGFBI, the M1 macrophage marker (CD86, iNOS) and M2 macrophage marker (CD163, CD206) expression in U937-derived M1 (M1) or M2 macrophages (M2). **(E)** The concentration of TGFBI in cells (M0, M1, M2) culture supernatants were assayed by ELISA. N = 3, **P < 0.01, ***P < 0.001, Student's t-test. **(F)** Representative immunofluorescent (IF) staining of TGFBI and the M2-like TAMs marker (CD163) in human GBM tissues. Areas indicated with solid and dashed square lines respectively represented the CD163 high and low expression groups. Scale bar represents 20 µm. **(G)** Quantification of the IF images (F) demonstrating abundant TGFBI expression in the CD163-enriched areas in human GBMs. N = 3, ****P < 0.0001, Student's t-test. **(H)** Representative IHC staining of TGFBI and CD163 in glioma microarray. Scale bar represents 100 µm. **(I)** Correlation analysis of TGFBI and CD163 expressions in glioma microarray (H), Pearson's r test. **(J)** Kaplan-Meier survival plot of TGFBI expression of glioma patients from the glioma microarray. P < 0.001, log-rank test. (K) Kaplan-Meier survival plot of TGFBI expression of GBM patients from CGGA cohort. P < 0.05, log-rank test.

M2-like TAMs, constituting the majority of macrophages in GBM 12, 39, fostered GSCs phenotype to mediate pro-tumor function via secreted mediators. To identify potential critical proteins secreted by M2-like TAMs, we screened three datasets, including GSE37475 (with 258 differentially expressed genes between M2^high^ TAMs and M2^low^ TAMs, Figure S2A) 15, genes encoding for secreted proteins and macrophage cell enhanced genes from the Human Protein Atlas (http://www.proteinatlas.org). The intersection of the three datasets comprised three genes (Figure 2A): AOAH, TGFBI, TIMP1. Additional survival analyses of GBM patients in TCGA database revealed that only TGFBI and TIMP1 expression were negatively associated with overall survival (Figure S2B), indicating their potential clinical significance in GBM progress. Next, glioma scRNA-seq datasets (GSE84465 40 and GSE148842 41) were analyzed to validate the relationship between TGFBI, TIMP1 and M2-like TAMs. T-distributed stochastic neighbor embedding (t-SNE) plots of GSE148842 and GSE84465 demonstrated that TGFBI was mainly expressed in TAMs (Figure 2B, Figure S2C), while TIMP1 was evenly expressed in almost all cell types (Figure S2C). We further explored their co-distribution with CD163, CD206 (markers of M2-like TAMs) in the two datasets. The t-NSE plots revealed that the co-distribution of TGFBI with CD163 and CD206 was much better than TIMP1 (Figure 2B right, Figure S2C). Meanwhile, the correlation analyses of TGFBI and CD163 in TCGA-GBM, CGGA-GBM, Rembrandt-GBM, Gravendeel-GBM databases suggested a strong positive correlation (Figure S2D). These bioinformatics analyses suggested that TGFBI was the potential molecular preferentially secreted by M2-like TAMs in the glioma microenvironment.

To validate the results from bioinformatics analysis, we investigated TGFBI expression in primed M0, M1, and M2 macrophages *in vitro* in polarized U937 monocytic cells to mimic TAMs as described 27, 33. Representative genes of M1 (iNOS, CD86) and M2 (CD163, CD206) macrophages were detected and polarization of U937 cells was validated (Figure S2E). The results indicated that TGFBI level was higher in M2 than M0 and M1 macrophages at RNA and protein levels (Figure 2C, 2D, and Figure S2E-S2H) and in cell culture supernatants as indicated by ELISA (Figure 2E). To further investigate if TGFBI was associated with M2-like TAMs *in vivo*, frozen human GBM sections were co-immunostained with CD163. The results showed that CD163 co-distributed with TGFBI, and high levels of CD163+ TAMs expressed more TGFBI (Figure 2F, 2G, and Figure S2I). TGFBI expression was markedly decreased in tumor regions lacking M2-like TAMs. Similarly, IHC analysis revealed a strong positive correlation between TGFBI and CD163 in glioma patients (Figure 2H and 2I). The Kaplan-Meier analysis of TGFBI expression in 76 gliomas indicated that higher expression denoted shorter survival time (Figure 2J). Significantly, higher TGFBI expression also informed poorer survival in CGGA-GBM patients (Figure 2K), indicating a potential pro-tumorigenic effect of TGFBI in promoting GBM malignancy. Collectively, these data demonstrated that TGFBI was preferentially secreted by M2-like TAMs and indicated poor prognoses in GBM patients.

### TGFBI is distributed around GSCs and mediates the pro-tumorigenic effect of M2-like TAMs

**Figure 3:**
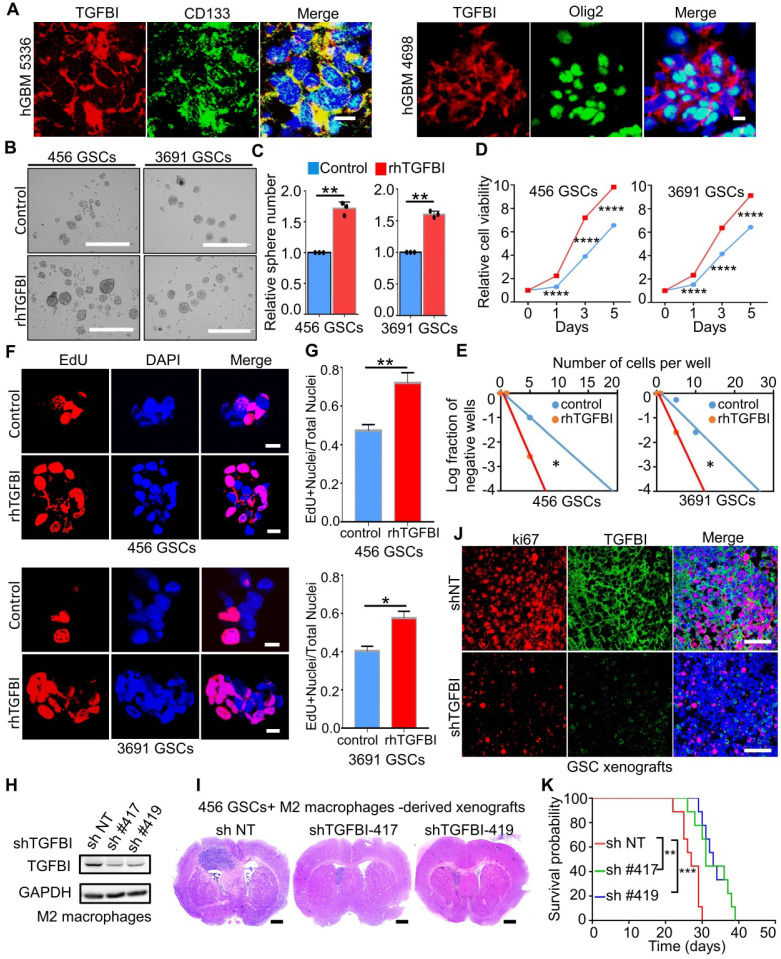
** TGFBI mediates the tumor**-**promoting effect of M2-like TAMs. (A)** Representative immunofluorescence (IF) staining of TGFBI and stem cell markers (CD133, Olig2) in human GBM were shown. Nuclei were counterstained with DAPI (blue). Scale bar represents 20 µm. **(B)** Representative tumorsphere images of GSCs (456 and 3691) cultured with rhTGFBI or control for 48 hours. Scale Bar: 400 µm. **(C)** Quantification of tumorspheres in (B). N = 3, **p < 0.01, Student's t-test. **(D, E)** Cell viability (D) and *in vitro* limiting dilution (E) assay of GSCs (456 and 3691) cultured with rhTGFBI or control. N = 6 biological independent samples. Data were represented as means ± SD, ****p < 0.0001, Student's t-test (D); *p < 0.05, likelihood ratio test (E). **(F)** Representative IF images of EdU incorporation. Scale bar represents 20 µm. **(G)** The quantification of the percentage of EdU+ cells. N = 3, *p < 0.05, **p < 0.01, Student's t-test. **(H)** IB analysis showed the extent of TGFBI knockdown in M2 macrophages. GAPDH was used as a loading control. **(I)** Representative HE staining of mouse brain (cross-section) 25 days after transplantation. Scale bar represents one cm. **(J)** Representative IF images of Ki67 in mouse models. Scale bar represents 200 µm. **(K)** Kaplan-Meier survival plot of mice co-implanted with GSCs (456) and shTGFBI M2 macrophages or shNT M2 macrophages. N = 9 for each group, **p < 0.01, ***p < 0.001, log-rank test.

Peripheral blood mononuclear cells could be recruited and re-educated into M2-like TAMs by GSCs to promote tumor enlargement 32. To determine whether TGFBI can mediate the tumor-promoting effect of M2-like TAMs, we studied the potential co-distribution between TGFBI and GSCs in human GBM frozen sections. Immunofluorescent staining of TGFBI and the GSC marker CD133, Olig2 demonstrated CD133+, Olig2+ cells were surrounded by abundant TGFBI (Figure 3A)**,** indicating their co-distribution. To further validate the function of TGFBI in GSC maintenance, we examined whether the exogenous recombinant human TGFBI (rhTGFBI) protein could promote GSC self-renewal and tumor growth. The addition of rhTGFBI increased the GSCs sphere number (Figure 3B-C), the cell viability (Figure 3D) and the fraction of GSCs able to form spheres (Figure 3E). EdU incorporation demonstrated rhTGFBI increased GSCs proliferation (Figure 3F-G).

Next, we investigated whether the knockdown of TGFBI could reduce the pro-tumorigenic effect of M2-like TAMs in GSC xenografts. Two distinct sets of non-overlapping small hairpin RNA (shRNA) were introduced to silence TGFBI in M2 macrophages, and its expression level was essentially lower than the non-targeting scramble shRNA (shNT, Figure 3H). We performed orthotopic GBM xenografts with co-injection of M2-macrophages expressing shTGFBI or shNT with the GSCs. When the first mouse of shNT showed neurologic signs, one mouse of each shTGFBI group was sacrificed for histological analysis. Haematoxylin and eosin staining demonstrated that shTGFBI mice harbored smaller tumor engraftments compared to shNT (Figure 3I). We then examined the proliferation of tumor cells from shNT and shTGFBI mice. Tumors from shTGFBI mice exhibited lower levels of proliferation (Ki67) (Figure 3J and Figure S3A-3B). IF staining with CD163 antibodies recognizes different species-derived antigens indicated the recruited macrophages (murine-derived TAMs) account for about 10% of the total macrophages (Figure 3J and Figure S3C-3D). Consequently, mice co-implanted with GSCs and shTGFBI M2-macrophages had significantly longer survival than those control mice (Figure 3K). Taken together, these data suggested that the M2-like TAMs-secreted TGFBI played an essential role in mediating the pro-tumorigenic effect of M2-like TAMs in GBMs.

### TGFBI could induce the reversion of differentiated GSCs

**Figure 4:**
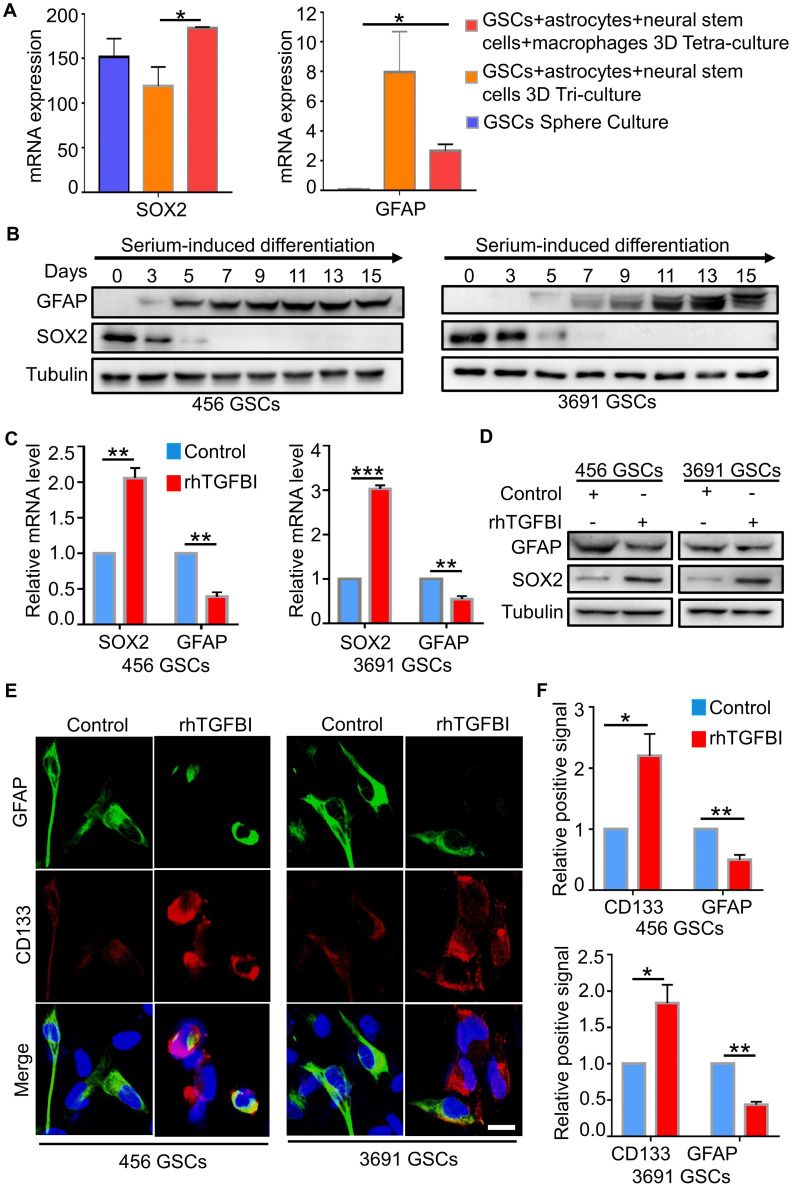
** TGFBI can de-differentiate differentiated GSCs. (A)** The mRNA level of GFAP and SOX2 in three different culture systems 45. *p < 0.05, Student's t-test for SOX2, ANOVA, analysis of variance for GFAP. **(B)** IB analysis of GFAP, SOX2 in GSCs (456 and 3691) cultured in serum-induced differentiation medium over a 15-day span. **(C, D, E)** qRT-PCR analyses (C), IB analyses (D), Representative IF images (E) of the GSC markers (CD133), the differentiation markers (GFAP) in GSCs (456 and 3691) cultured with rhTGFBI or control. Scale bar represents 20 µm. N = 3, **P < 0.01, ***p < 0.001, Student's t-test. **(F)** The quantification of IF images (E) showed DGCs with rhTGFBI stimulation re-expressed of CD133 and decreased in GFAP content. N = 3, *p < 0.05, **P < 0.01, Student's t-test.

GSCs exhibit cellular plasticity, displaying that differentiated GSCs can acquire GSCs stemness due to the microenvironment alteration 42-44. The tetra-culture model, including macrophages, demonstrated an upregulated GSC signature and a downregulated differentiated glioblastoma cells (DGCs) signature compared to the tri-culture model without macrophages (Figure 4A) 45. To investigate whether M2-like TAMs-secreted TGFBI could induce the reversion of differentiated GSCs, we induced *in vitro* differentiation of GSCs by withdrawing growth factors and adding serum-containing medium (DMEM with 10% FBS) 32. After five days of serum induce, these cells displayed the differentiation marker GFAP with disappeared stemness marker SOX2, indicating successful differentiation (Figure 4B). Next, we applied rhTGFBI (10 ug/ml) into the medium of differentiated GSCs for 48 hours. Analysis of the corresponding markers showed that the cells with rhTGFBI stimulation re-expressed SOX2, CD133 and decreased in GFAP content (Figure 4C-F). Collectively, these data revealed M2-like TAMs-derived TGFBI could induce differentiated GSCs back to GSC-like cells.

### Integrin αvβ5 is a receptor for TGFBI on GSCs

**Figure 5:**
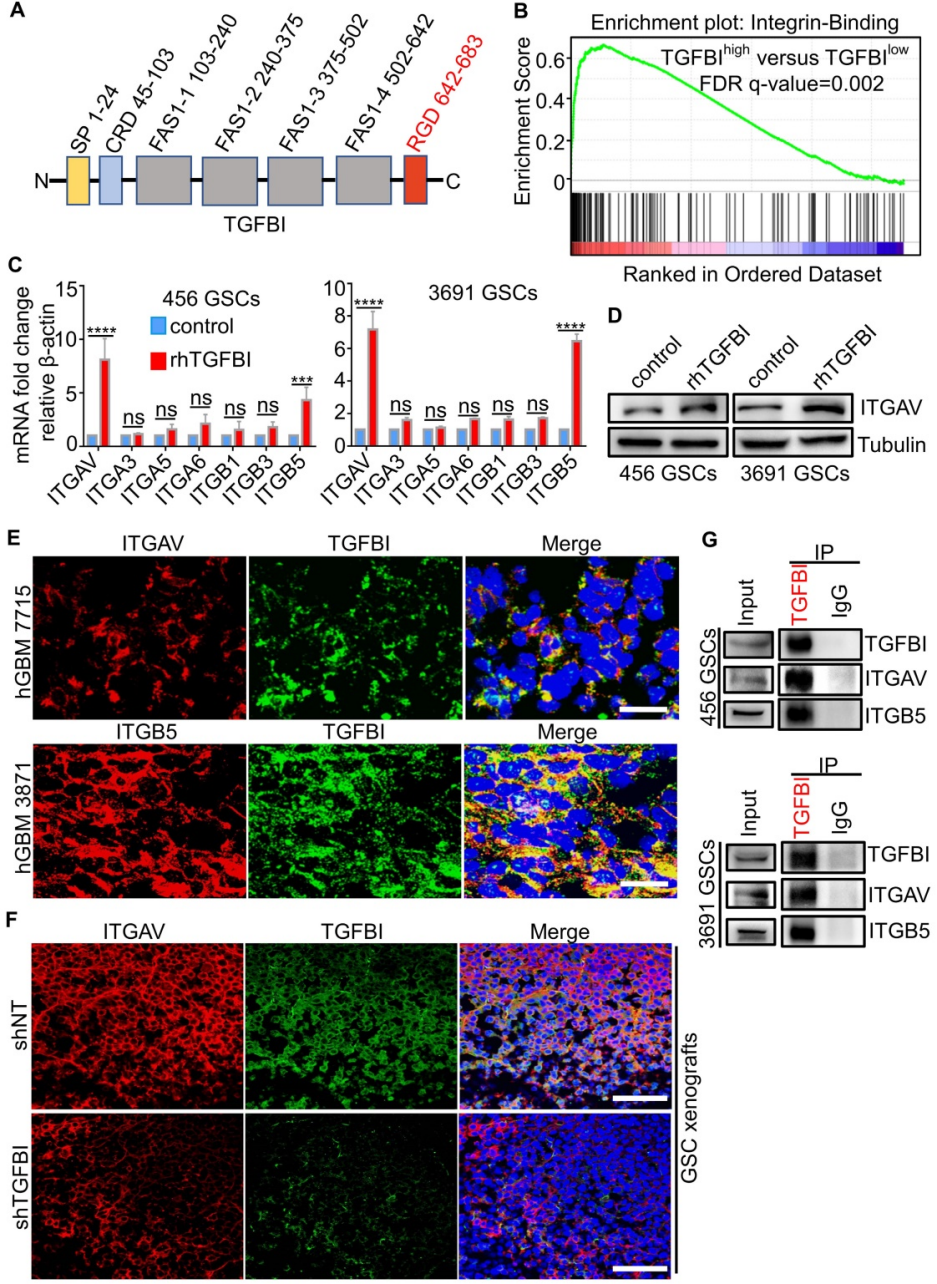
** TGFBI functions via binding to integrin αvβ5 on GSCs. (A)** Schematic diagram of TGFBI protein structure. It contained a secretory signal (SP) in the N-terminal cysteine-rich domain (CRD), followed by four fasciclin-1 domains (FAS1 1-4) and Arg-Gly-Asp (RGD) in the C-terminal. The RGD motif could bind the integrins. **(B)** GSEA of the TGFBI high expression versus low expression in TCGA GBM RNA-seq indicated the integrin-binding pathway was significantly upregulated in the TGFBI high expression group. **(C)** qRT-PCR analyses of the expression of the αv, α3, α5, α6, β1, β3, and β5 in GSCs (456 and 3691) cultured with rhTGFBI or control. N = 3, ***P < 0.001, ****P < 0.0001, ANOVA, analysis of variance. **(D)** IB analysis of ITGAV in GSCs (456 and 3691) cultured with rhTGFBI or control. **(E)** Representative immunofluorescence (IF) staining of TGFBI and the potential receptors (ITGAV and ITGB5) in human GBM were shown. Scale bar represents 20 µm. **(F)** Representative IF images of ITGAV and TGFBI in mouse models. Scale bar represents 200 µm. **(G)** Co-immunoprecipitation of ITGAV and ITGB5 with the TGFBI-specific antibody from 456 and 3691 GSC cell lysates. Immunoglobulin G (IgG) was used as a control antibody for IP.

To investigate the molecular mechanisms that mediate the pro-tumorigenic effect of TGFBI on GSCs, we attempt to determine the receptor for TGFBI. The protein structure of TGFBI includes Arg-Gly-Asp (RGD) motifs in the C-terminus, known as integrin-binding motifs (Figure 5A) 28, 29. Previous reports indicated TGFBI exerted biological function via binding the integrins located on the cellular surface in other cancers 46-48. We performed GSEA of the TGFBI^high^ expression versus TGFBI^low^ expression in TCGA-GBM RNA-seq and found the integrin-binding pathway was significantly upregulated in the TGFBI^high^ group (Figure 5B). Integrins containing two subunits of α and β, αv 49, α3 50, α5 51, α6 52, β1 53, β3 51, and β5 49 were known to be expressed explicitly in GSCs and maintained the GSCs stemness and tumor growth. To explore the specific subunits mediating the signal of TGFBI on GSCs, we examined changes in RNA expression of the above integrins using qRT-PCR after GSCs stimulated with rhTGFBI. Integrin αv (ITGAV) and β5 (ITGB5) were the most significantly upregulated in GSCs (456 and 3691) (Figure 5C), of which ITGAV was validated by immunoblot (Figure 5D). IF staining demonstrated TGFBI and ITGAV, ITGB5 co-located on GSCs membrane in human GBM samples (Figure 5E) and mouse xenografts samples (Figure 5F). Compared to shTGFBI tumors, the fluorescence signal of ITGAV and TGFBI in shNT group was much stronger (Figure 5F). Furthermore, we verified the interaction between TGFBI and ITGAV, ITGB5 by co-immunoprecipitation with the anti-TGFBI antibody (Figure 5G). The correlation between TGFBI and ITGAV, ITGB5 were strongly positive in mRNA level in TCGA-GBM (Figure S4A) and CGGA-GBM cohorts (Figure S4B). Meanwhile, integrin αv, β5 were significantly overexpressed in the TCGA-GBM sample compared to the normal brain (Figure S4C). GBM patients with a high level of both ITGAV and ITGB5 indicated a shorter survival time (Figure S4D). Taken together, these data suggest that integrin αvβ5 is the receptor mediating the signal of TGFBI on GSCs.

### TGFBI signals via integrin αvβ5-Src-Stat3 to function the pro-tumorigenic effect

**Figure 6:**
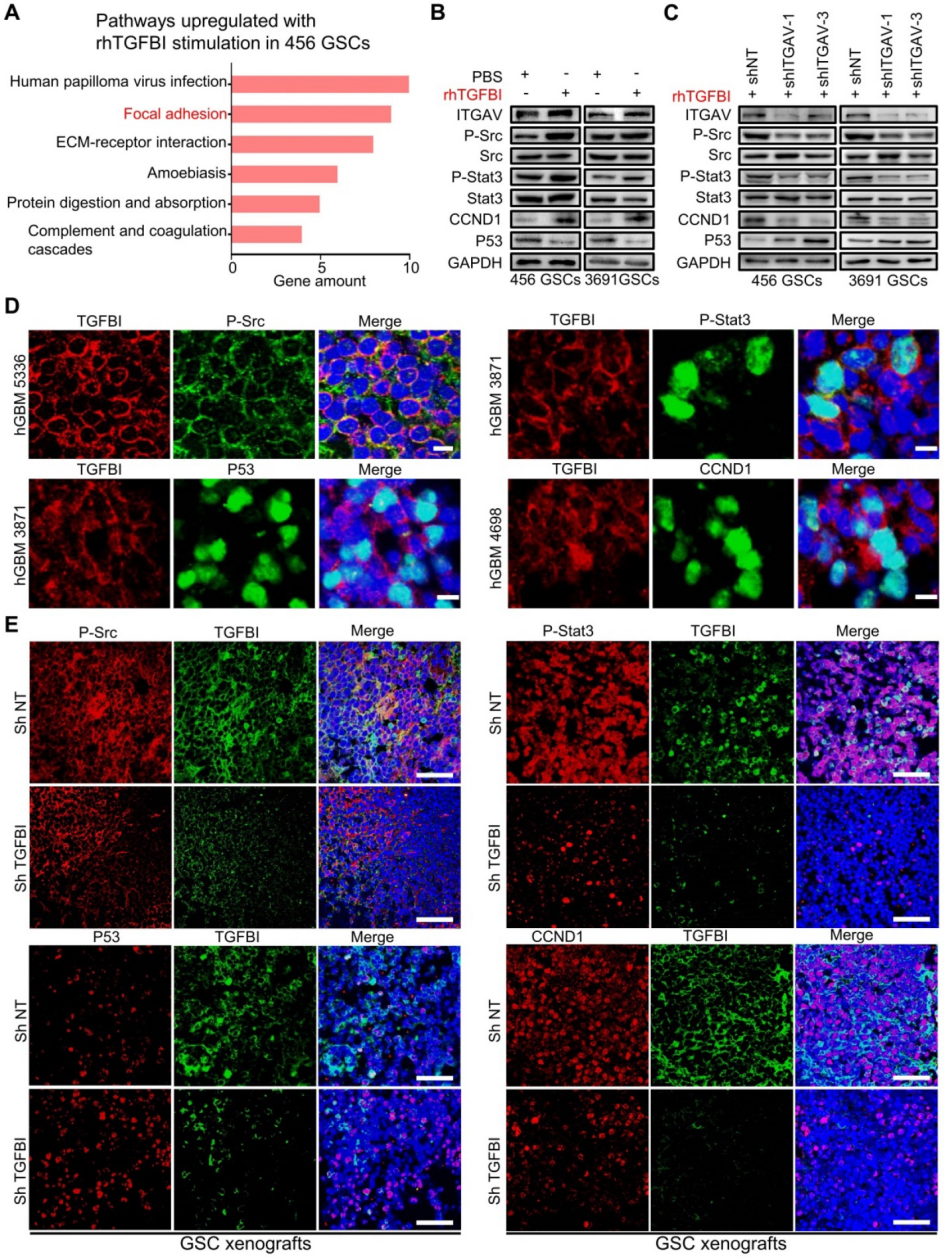
** TGFBI mediates the pro-tumorigenic effect via the integrin αvβ5-Src-Stat3 axis. (A)** KEGG pathway enrichment analysis of upregulated genes (fold change ≥ 2.0) in the rhTGFBI stimulation group versus the control group (456 GSCs). **(B, C)** Immunoblot analyses of phospho-Src (P-Src-Y527), total Src, phospho-Stat3 (P-Stat3-Y705), total Stat3, P53 and CCND1, indicated that rhTGFBI stimulation significantly increased Src and Stat3-activating phosphorylation (B), while the knockdown ITGAV treatment compromised rhTGFBI-stimulated Src and Stat3 activation in GSCs (C). **(D)** IF stainings of TGFBI and P-Src (upper left), P-Stat3 (upper right), P53 (bottom left), CCND1 (bottom right) in human GBMs. The scale bars measure 10 µm. Nuclei were counterstained with DAPI (blue). **(E)** IF stainings of TGFBI and P-Src (upper left), P-Stat3 (upper right), P53 (bottom left), CCND1 (bottom right) in GBM xenografts with shNT or shTGFBI. Nuclei were counterstained with DAPI (blue). The scale bars measure 200 µm.

To further delineate the downstream pathways mediating the TGFBI- integrin αvβ5 signaling in GSCs, we performed Kyoto Encyclopedia of Genes and Genomes (KEGG) pathway enrichment analysis of upregulated genes (fold change ≥ 2.0) in the rhTGFBI stimulation group versus the control group (Figure 6A). The results indicated that the focal adhesion pathway was one of the two most clearly affected with rhTGFBI stimulation. Consistently, the focal adhesion and JAK-STAT signaling pathways were also enriched in TGFBI^high^-ITGAV^high^-ITGB5^high^ group in the TCGA-GBM cohort (Figure S5A). TGFBI had been found to promote metastasis via integrin αvβ5-Src axis in human colon cancer cells 54. Phosphorylation of Stat3 in human hepatocellular carcinoma stem cells mediated the promote self-renewal effect of TAMs 55. Simultaneously, the protein-protein interaction (PPI) network built using STRING (http://string-db.org/) indicated that the TGFBI-integrin αvβ5-Src-Stat3 axis might be a functional protein association network in GSCs (Figure S5B). We then validated P-Src and P-Stat3 were upregulated in GSCs after rhTGFBI stimulation via immunoblotting assay, whereas knockdown of ITGAV attenuated their activation (Figure 6B-6C). The downstream targets of P-Stat3, CCND1 and P53, changed correspondingly (Figure 6B-6C). When the inhibitor of ITGAV and ITGB5 (SB273005) was applied in GSCs, the activation of P-Src and P-Stat3 by rhTGFBI was inhibited compared to the control group (Figure S5C).

We next evaluated the expression of TGFBI and P-Src, P-Stat3, CCND1, and P53 in three different human GBM specimens. By IF co-staining, TGFBI co-expressed with P-Src and P-Stat3 (Figure 6D) and also co-expressed with the P-Stat3 downstream targets CCND1 and P53 (Figure 6D). We further assessed their expression in orthotopic models. Compared to shNT tumors, the fluorescence signal of P-Src and P-Stat3 in tumors with TGFBI knockdown was much weaker (Figure 6E). As expected, the fluorescence signal of CCND1 and P53 changed correspondingly (Figure 6E). Collectively, these data showed that the TGFBI-integrin αvβ5 binding activated Src phosphorylation, thus upregulating the Stat3 pathway for the pro-tumorigenic effect.

### TGFBI could be used as a potential bio-index in the assessment of GBM diagnosis and progression

**Figure 7:**
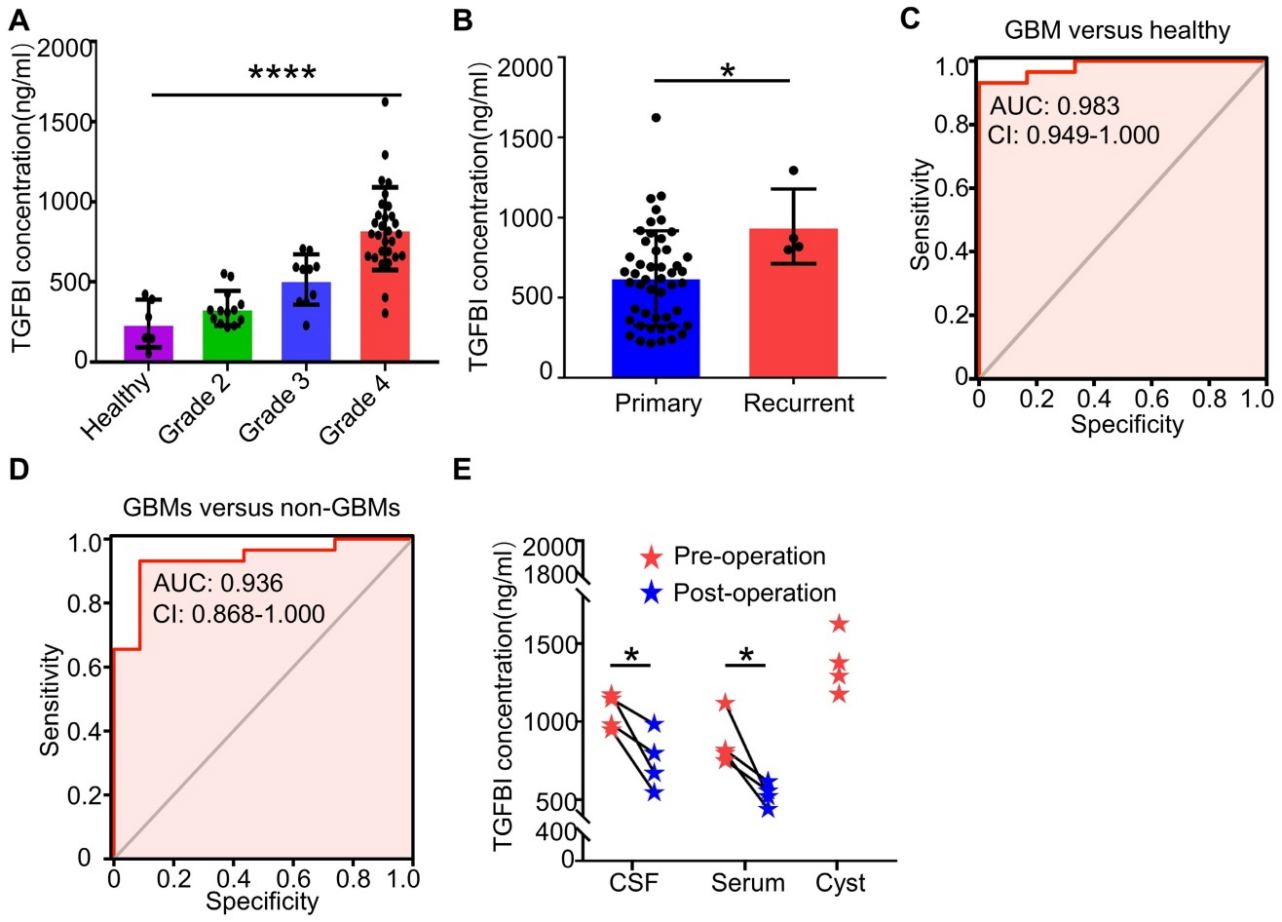
** TGFBI could be used as a potential bio**-**index in the assessment of GBM diagnosis and progression. (A)** Serum TGFBI concentrations in six healthy controls and 52 glioma patients (grade 2 (n = 13), grade 3 (n = 10), grade 4 (n = 29)). ****p < 0.0001, ANOVA, analysis of variance. **(B)** Serum TGFBI concentrations in 48 primary gliomas and four recurrent gliomas. *p < 0.05, Student's t-test. **(C, D)** Receiver-operator characteristic (ROC) analyses of TGFBI concentrations between GBM patients and healthy controls (C), non-GBM patients (WHO grade 2 and grade 3) (D) respectively exhibited an AUC of 0.983 and 0.936. **(E)** The dot plots show the changes in the CSF, serum values for TGFBI before and seven days post-resection for the four cystic GBM patients. The intro-operative sac fluid was also assayed for TGFBI concentration. *P < 0.05, Student's t-test.

TGFBI had been reported to exhibit higher serum levels in brain tumor patients compared to non-tumor patients 46. To investigate its serum range in glioma patients and the healthy cohort, we assayed serum TGFBI levels in 6 healthy controls and 52 glioma patients. Of which four cystic GBM patients, the preoperative serum and cerebrospinal fluid (CSF), intraoperative sac fluid, serum and CSF TGFBI concentrations on the seventh day after surgery were also assayed. Identical collection and preservation procedures were utilized for all samples. The results showed serum TGFBI concentrations of GBMs were much higher than healthy controls, WHO grade 2 and grade 3 gliomas (Figure 7A). In recurrent gliomas, the mean protein level of TGFBI was higher than in primary gliomas (Figure 7B). Receiver-operator characteristic (ROC) analyses of TGFBI concentrations between GBM patients and healthy controls exhibited an area under the curve (AUC) of 0.983 (Figure 7C). And the AUC of GBMs versus non-GBMs (WHO grade 2 and WHO grade 3) was 0.936 (Figure 7D). Furthermore, of the four cystic GBM patients, sac fluid contained more TGFBI than preoperative-serum and CSF (Figure 7E). Interestingly, the postoperative TGFBI concentration in serum and CSF decreased significantly (Figure 7E). Taken together, these data suggested TGFBI could be used to assess the diagnosis and progression of GBM.

## Discussion

GBM exhibits striking cellular heterogeneity, with abundant TAMs in the tumor microenvironment 9. Previous research indicated that high TAMs accumulation in gliomas was associated with an unfavorable prognosis 12. Consistently, we integrated eight marker genes of M2-like TAMs to construct a gene signature for M2 TAMs and confirmed the signature was positively correlated with WHO grades and indicated dismal survival. However, the exact molecular mechanisms underlying TAMs' functions in promoting GSC maintenance and GSC-driven tumor growth remain largely unknown, except for a few studies reporting molecules, e.g., TGFβ1 18 and PTN 27 were specifically involved. In this study, we uncovered that M2-like TAMs secreted the extracellular matrix protein TGFBI to enhance GSCs stemness characteristics and GBM progression. Knocking down TGFBI by shRNA largely abrogated the tumor-supportive effect of TAMs compared to shNT. Increased TAM-derived TGFBI has been found in ovarian cancer and is related to tumor growth and dismal prognosis 56, 57. TGFBI secreted by TAMs in ovarian cancer contributed to an immunosuppressive TME and promoted cell migration, and meanwhile, anti-TGFBI antibody treatment reduced peritoneal tumor size in the orthotopic mouse model 56, 57. In gastrointestinal tract cancers and urothelial carcinomas, TGFBI also facilitated cell proliferation and migration, although the source of TGFBI in these cancers was found to be tumor cell derived 31, 46, 54, 58. Our results indicated the critical role of TGFBI secreted by M2-like TAMs in GSC-driven glioma growth, adding credence to the importance of TGFBI across cancers.

By protein domain analysis and co-immunoprecipitation assay, we identified integrin αvβ5 as the membrane receptor mediating TGFBI signaling in GSCs. In pancreatic and colon cancers, tumor cell-derived TGFBI has also been reported to function via binding to integrin αvβ5 54, 58. Notably, integrin αvβ5 was deemed as a functional GSC marker essential for GBM maintenance, and specifically mediated infection of oncolytic virus Zika in GSCs. Meanwhile, targeting integrin αvβ5 attenuated GSC viability and self-renewal, reducing GSC-driven tumor growth in mice 59. Our results suggested TGFBI as an active ligand of integrin αvβ5 mediating M2-like TAMs functions on GSCs, and binding of TGFBI to integrin αvβ5 on GSCs increased phosphorylation of the tyrosine kinase Src, with elevated phosphorylation of Stat3. Consistently, the integrin-Src-Stat3 pathway has been reported to be involved in maintaining stemness and promoting the proliferation of cancer stem cells in various tumors 60-66. Noteworthy, although Src-Stat3 is the main downstream pathway activated, integrin αvβ5 also functions as a cellular membrane receptor on a wide range of substrates that activate multiple signaling and influence additional cellular activities.

Targeting the molecular link mediating TAMs pro-tumor effect on glioma cells represents an attractive therapeutic strategy. By secreting various factors and affecting other immune cells, TAMs have been demonstrated to be involved directly or indirectly in promoting multiple aspects of tumorigenesis 67. Therapeutic strategies based on or combined with TAMs have the potential to improve the treatment efficacy of cancer therapies, and the strategies can be broadly divided into reducing the number of TAMs or altering their functionality within the tumor microenvironment 68. Notably, Pyonteck SM et al. found CSF1/CSF1R blockade, the most widely studied axis for TAM depletion and reducing tumor volume in several xenograft models 15, blocked glioma growth and progression through a mechanism in which TAMs were not depleted but were instead 're-educated' within the glioma microenvironment 15. Given the dynamics and plasticity of TAMs phenotypes during tumor progress and recurrence 15, 67, targeting the paracrine factors secreted by TAMs, instead of TAMs themselves, may be a more actionable strategy. TGFBI, specifically secreted by TAMs and presented very low in GBM cells as analyzed using the single-cell sequencing data (Figure 2B and Figure S2C), constitutes such an ideal target, and significantly its expression remains high from the primary to recurrent tumors (Figure 7B).

Identifying diagnostic and prognostic markers for GBM in more accessible specimens such as blood and/or CSF would help clinical treatment decision-making and monitoring. A few proteins of parenchymal glioma origin have been reported to be detected in patient blood and are of clinical relevance 69-71. The proteins include GFAP, YKL‑40 and IGFBP-2, which are either cytoskeletal proteins in astroglial tumors, or overexpressed genes of the tumor cells that may be involved in tumorigenesis. Among these proteins of glioma cell origin, only YKL-40 is found to be also secreted by TAMs 71. Different from these markers, TGFBI is secreted by TAMs and very low in GBM cells. Together with its critical role in enhancing glioma growth, TGFBI level in patient serum/CSF may distinctly index TAM abundance and its functionality in glioma progress. Our clinical data accordingly demonstrates a positive correlation of the serum and CSF TGFBI levels with tumor malignancy and tumor burden of glioma patients. Therefore, we are about to further confirm whether serum TGFBI is an early warning index for GBM recurrence. Expanded longitudinal studies of the serum and CSF TGFBI level measured prospectively in glioma patients are warranted for its potential application as a glioma circulating marker.

In summary, our data demonstrated that TGFBI was secreted by M2-like TAMs, promoting the maintenance of GSCs and glioma growth through integrin αvβ5-Src-Stat3 signaling. Disruption of TGFBI-integrin αvβ5 signaling attenuated the tumor-promoting effect of TAMs and extended mice survival time, indicating this pathway as an attractive therapeutic target for GBM. In addition, high serum or CSF TGFBI may serve as a potential diagnostic and prognostic bio-index for GBMs.

## Supplementary Material

Supplementary figures and tables.

## Abbreviations

GBM: glioblastoma
GSCs: glioblastoma stem cells
TAMs: tumor-associated macrophages
TGFBI: transforming growth factor beta-induced
FBS: fetal bovine serum
IF: immunofluorescence
IHC: immunohistochemistry staining
EdU: 5-Ethynyl-2′-deoxyuridine
qRT-PCR: quantitative real-time PCR
ELISA: enzyme-linked immunosorbent assay
TCGA: The Cancer Genome Atlas
CGGA: Chinese Glioma Genome Atlas
GEO: Gene Expression Omnibus
TMA: Tissue microarray
scRNA-seq: single-cell RNA-sequencing
ssGSEA: single-sample Gene set enrichment analysis
DGCs: differentiated glioblastoma cells
ITGAV: Integrin αv
ITGB5: Integrin β5
KEGG: Kyoto Encyclopedia of Genes and Genomes
ROC: Receiver-operator characteristic
AUC: area under the curve
