# Supplementary material for: TGFBI secreted by tumor-associated macrophages promotes glioblastoma stem cell-driven tumor growth via integrin αvβ5-Src-Stat3 signaling
Source: Theranostics. 2022 May 16;12(9):4221–36. doi: 10.7150/thno.69605 (PMC9169371; doi:10.7150/thno.69605)
Supplement: Supplementary file 1 — Supplementary figures and tables. [file thnov12p4221s1.pdf]

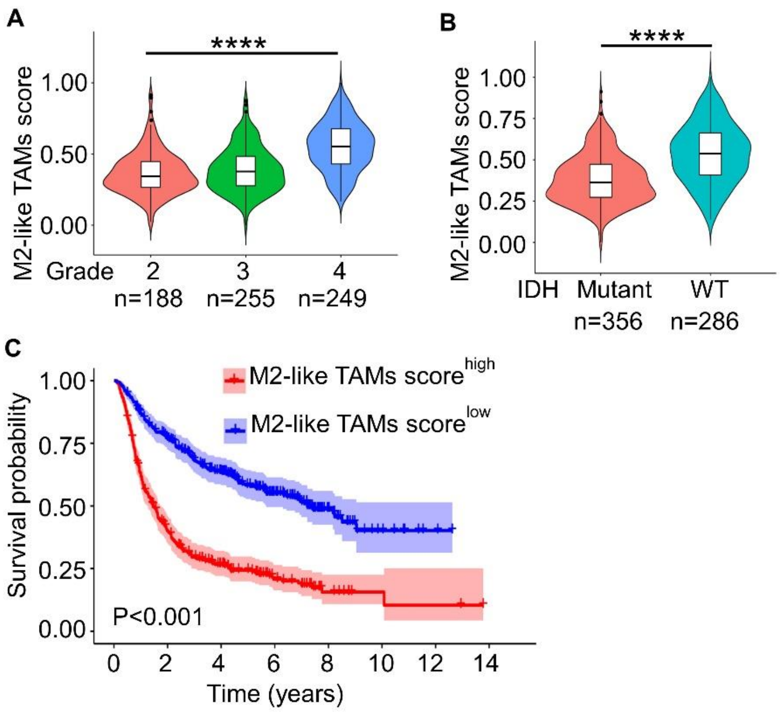

Figure S1. M2-like TAMs infiltrate in CGGA cohort.

(A) ssGSEA of M2-like TAMs signature genes in CGGA-glioma dataset showed M2-like TAMs signature score increased with higher grade gliomas. ****P < 0.0001, ANOVA, analysis of variance. (B) Violin plot of M2-like TAMs signature score in IDH-WT and IDH-mutant gliomas from the CGGA database. ****P < 0.0001, Student's t-test. (D) Kaplan–Meier survival plots of M2-like TAMs signature score showed a higher score indicated a poorer prognosis. P < 0.001, log-rank test.

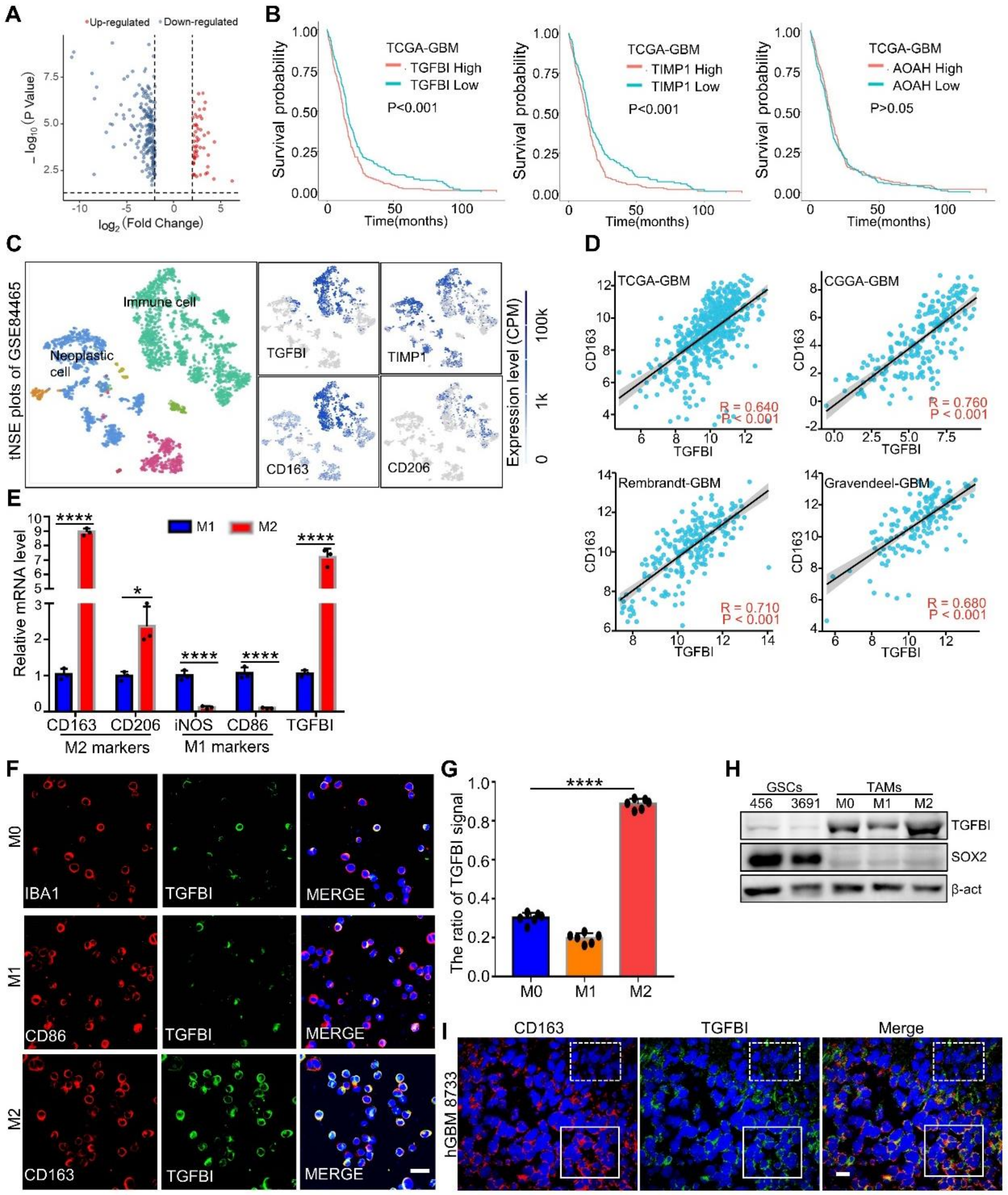

Figure S2. TGFBI is preferentially secreted by M2-like TAMs and indicates a poor prognosis in GBM patients.

(A) The volcano plot shows the differentially expressed genes in GSE37475 (fold change ≥ 2) [1]. (B) Kaplan- Meier survival plots of TGFBI, TIMP1, AOAH of TCGA-GBM patients. Statistical test: Log-rank test. (C) t-SNE analysis of 3533 cells of 4 glioma patients (GSE84465) [2]. Differential coloring in cell clusters is annotated according to the dominant cell type (left). Expression of cell-type-specific TGFBI, TIMP1, CD163 and CD206 overlaid on the t-SNE space (right). (D) Correlation analyses of TGFBI and CD163 in TCGA-GBM, CGGA-GBM, Rembrandt-GBM, Gravendeel-GBM databases. Pearson's r test. (E) qRT-PCR analyses of the expressions of M2 markers (CD163 and CD206), M1 markers (iNOS and CD86) and TGFBI between primed-U937 and the U937-derived M2-like TAMs. *p < 0.05, ****p <

0.0001, Student’s t-test. (F) Immunofluorescent staining of TGFBI and IBA1, CD86, CD163 in M0, M1 and M2 macrophages. Scale bar represents 50 μm. (G) The quantification of the TGFBI signal is shown. ****$p < 0.01$, ANOVA, analysis of variance. (H) Immunoblot analysis of TGFBI in GSCs (456 and 3691) and TAMs. (I) Representative immunofluorescent (IF) staining of TGFBI and the M2-like TAM marker (CD163) in human GBM tissues. Areas indicated with solid and dashed square lines respectively represent the CD163 high and low expression groups. Scale bar represents 20 μm.

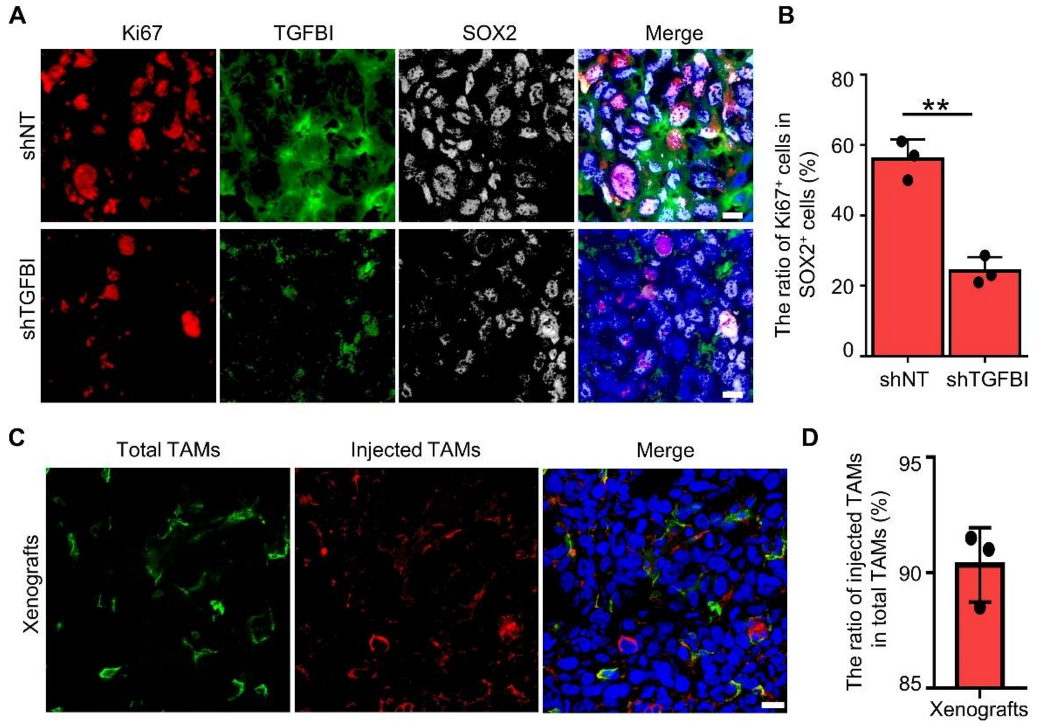

Figure S3 TGFBI is distributed around GSCs and mediates the pro-tumorigenic effect of M2-like TAMs.

(A) Representative IF images of Ki67, TGFBI, and SOX2 in mouse models. Scale bar represents 10 μm. (B) The ratio of $Ki67^+$ cells in $SOX2^+$ cells. n = 3, **$p < 0.01$, Student's t-test. (C) Representative IF images of total TAMs (labeled with CD163 antibody recognizes human and murine-derived antigen) and injected TAMs (labeled with CD163 antibody recognizes human-derived antigen) in mouse models. Scale bar represents 20 μm. (D) The statistical result indicated the injected TAMs (human-derived TAMs) account for about 90% of the total TAMs (human and murine-derived TAMs), n = 3.

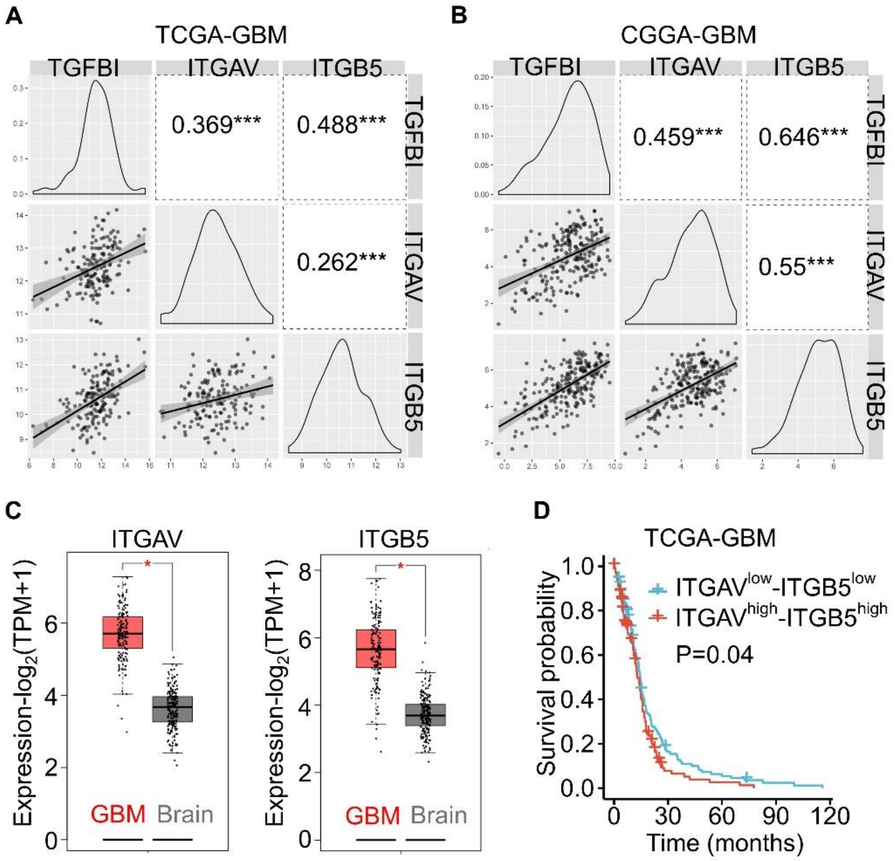

Figure S4 Integrin αvβ5 is a receptor for TGFBI on GSCs.

(A, B) The correlation analyses of ITGAV, ITGB5, and TGFBI expression in TCGA-GBM (A) and CGGA-GBM (B) databases. ***$p < 0.001$, Pearson's r test. (C) The mRNA levels of ITGAV and ITGB5 between matched TCGA GBM and normal brain from the genotype-tissue expression (GTEx) dataset. *$p < 0.05$, Student's t-test. (D) Kaplan- Meier survival plots of ITGAV and ITGB5 of TCGA-GBM patients. P=0.04, log-rank test.

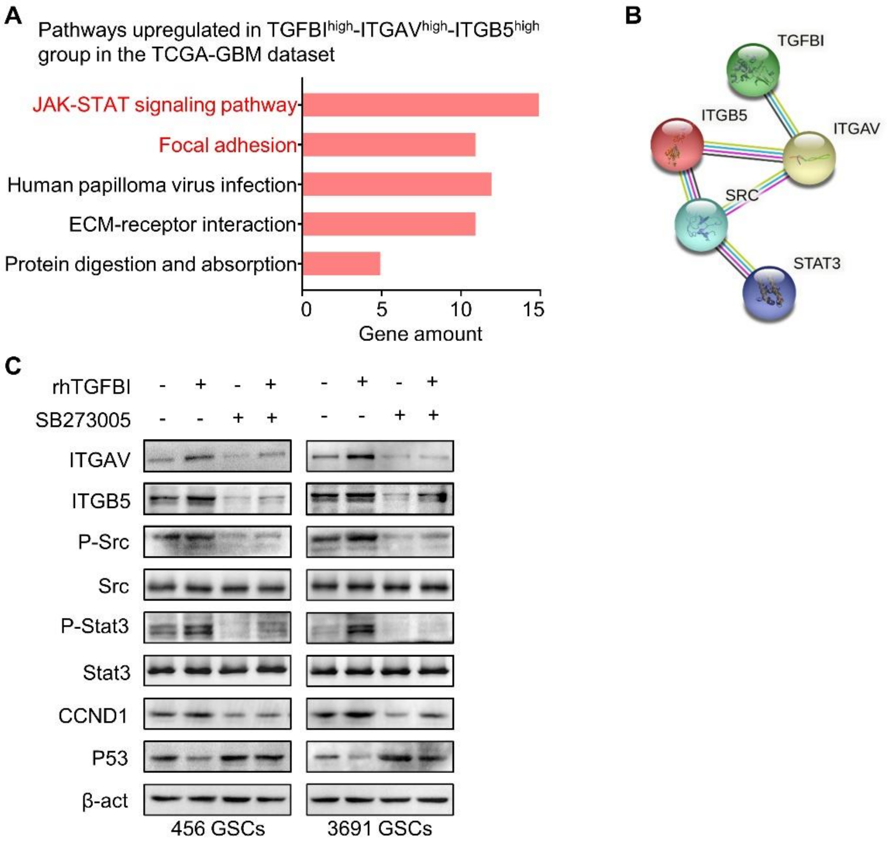

Figure S5. TGFBI mediates the pro-tumorigenic effect via the integrin αvβ5-Src-Stat3 axis.

(A) KEGG pathways were enriched with TGFBI^{high}-ITGAV^{high}-ITGB5^{high} in TCGA-GBM. (B) The protein-protein interaction (PPI) network of TGFBI-ITGAV-ITGB5-Src-Stat3 was built using STRING (http://string-db.org/). (C) Immunoblot analyses of ITGAV, ITGB5, phospho-Src (P-Src-Y527), total Src, phospho-Stat3 (P-Stat3-Y705), total Stat3, P53 and CCND1 in 456GSCs and 3691GSCs, indicated that rhTGFBI stimulation significantly increased Src and Stat3-activating phosphorylation, while the inhibitor of ITGAV and ITGB5 (SB273005) compromised rhTGFBI-stimulated P-Src and P-Stat3 activation in GSCs.

Table S1 The list of chemicals.

**Chemicals**	**Source**	**Identifier**
PMA	Sigma-Aldrich	P1585
LPS	Sigma-Aldrich	L4516
IFN-γ	Peprotech	AF300-02
IL4	Peprotech	AF200-04
IL10	Peprotech	AF200-10
TGF-β	Peprotech	AF100-21C
EGF	R&D	Cat#236-EG
bFGF	R&D	Cat#4114-TC
Fetal bovine serum	Gbico	10099141C
protease and phosphatase inhibitors	Thermo Scientific	Cat# 78442
ChamQ SYBR Master Mix	Vazyme	Cat# Q311-02/03
HiScript II Q RT SuperMix for qPCR (+gDNA wiper)	Vazyme	Cat# R223-01
B27	BasalMedia	S441J7
Neurobasal media	BasalMedia	X087G1
Sodium pyruvate	BasalMedia	Cat# 11360070
Glutamax	BasalMedia	Cat# 35050061
penicillin/streptomycin	BasalMedia	S110JV
RPMI Medium 1640 basic	Gbico	Cat# 11875093
Triton X-100	Solarbio	Cat# T8200
SB273005	Selleck	S7540

Table S2 The clinical information of the six human GBM specimens.

**Specimen**	**Histopathology**	**WHO Grade**	**Gender**	**Age (years)**	**Predominant side of tumor location**	**Predominant lobe of tumor location**	**Extent of surgical resection**
GBM5336	GBM	4	Male	64	Left	Temporal	TR
GBM3871	GBM	4	Female	49	Right	Temporal	TR
GBM4698	GBM	4	Female	46	Left	Temporal	TR
GBM7611	GBM recurrent	4	Female	56	Right	Parietal	GTR
GBM8733	GBM	4	Male	54	Left	Temporal	TR
GBM7715	GBM recurrent	4	Female	45	Right	Frontal	TR

Abbreviations: GBM, Glioblastoma; TR, Total resection; GTR, Gross total resection.

Table S3 The clinical information of the 78 gliomas.

TMA number	Gender	Age (year)	WHO grade	Survival time (month)	Survival state
1	Male	63	2	13.32	Dead
2	Male	47	4	4.80	Dead
3	Male	33	3	21.37	Dead
4	Male	44	4	NA	NA
5	Female	73	4	42.08	Alive
6	Male	55	4	9.24	Dead
7	Male	63	4	5.92	Dead
8	Male	58	4	8.52	Dead
9	Female	47	2	36.36	Alive
10	Female	44	2	36.07	Alive
11	Male	65	4	15.78	Dead
12	Male	68	4	18.12	Dead
13	Male	35	4	NA	NA
14	Female	42	2	35.38	Alive
15	Male	30	4	35.34	Alive
16	Female	35	2	34.49	Alive
17	Female	53	2	34.49	Alive
18	Male	48	4	1.35	Dead
19	Male	47	2	34.19	Alive
20	Female	44	2	34.16	Alive
21	Male	46	2	29.36	Dead
22	Female	43	3	NA	NA
23	Male	60	4	13.87	Dead
24	Male	65	4	11.51	Dead
25	Female	56	3	13.48	Dead
26	Female	50	2.3	NA	NA
27	Female	50	4	NA	NA
28	Male	50	4	NA	NA
29	Female	42	4	6.64	Dead
30	Male	45	3	28.77	Alive
31	Male	44	4	13.18	Dead
32	Male	46	4	28.47	Alive
33	Female	51	4	9.40	Dead
34	Male	65	4	16.34	Dead
35	Male	62	4	14.50	Dead
36	Female	36	2	27.48	Alive
37	Female	35	4	18.67	Dead
38	Male	30	4	NA	NA
39	Male	65	4	6.90	Dead
40	Male	11y5m	4	10.75	Dead

42	Female	26	4	24.36	Dead
43	Male	43	4	2.89	Dead
44	Male	61	4	9.53	Dead
45	Female	38	2	24.72	Alive
46	Female	38	4	21.80	Alive
47	Female	54	4	16.93	Dead
48	Male	46	4	23.61	Alive
49	Female	48	3	2.73	Dead
50	Male	31	2	18.87	Alive
51	Female	47	3	NA	NA
52	Male	55	4	18.31	Alive
53	Female	37	2.3	17.62	Alive
54	Male	53	4	30.31	Alive
55	Male	47	2	35.34	Alive
56	Male	31	2	34.26	Alive
57	Male	49	4	13.78	Alive
58	Female	50	4	18.77	Alive
59	Female	9	3	15.81	Alive
60	Male	46	4	10.45	Dead
61	Female	62	4	10.78	Dead
62	Male	1y6m	3.4	21.14	Alive
63	Female	32	2.3	0.33	Alive
64	Female	55	4	6.87	Dead
66	Male	34	2	23.21	Alive
67	Female	34	2	2.86	Dead
68	Male	69	4	11.64	Dead
69	Female	57	4	NA	NA
70	Male	17	3	NA	NA
71	Male	45	2	NA	NA
73	Female	55	3.4	3.68	Dead
74	Female	59	2.3	13.94	Dead
75	Male	48	2	NA	NA
76	Male	52	2	16.14	Dead
77	Male	40	2	31.96	Alive
78	Male	56	4	4.83	Dead
79	Female	61	2	NA	NA
80	Male	1y8m	2	26.89	Alive
81	Male	16	3	NA	NA

Abbreviations: NA, Not available

Table S4 The list of antibodies.

**Antibodies**	**Source**	**Identifier**
P-stat3 Y705	Cell Signaling	9145S
Stat3	Cell Signaling	9139S
P-src Y527	Cell Signaling	2105S
Src	Cell Signaling	2109S
CCND1	Cell Signaling	2978T
Ki67	Proteintech	27309-1-AP
P53	Santa cruz	SC126(D0-1)
TGFBI	Proteintech	60007-1-Ig
TGFBI	Proteintech	10188-1-AP
TGFBI	Abclone	A11222
TGFBI	Abcam	ab170874
CD206	Proteintech	60143-1-Ig
CD163	Santa cruz	SC-33715
CD163	Proteintech	16646-1-AP
IBA1	Cell Signaling	17198S
ITGAV	Abclone	A2091
ITGB5	Cell Signaling	3629S
SOX2	Proteintech	66411-1-Ig
Olig2	Proteintech	66513-1-Ig
CD133	Affinity	BF0403
GFAP	Cell Signaling	80788
CD86	Proteintech	13395-1-AP
iNOS	Proteintech	18985-1-AP
SOX2	Abcam	ab196637

Table S5 Short hairpin RNA sequences used for lentiviral vector construction

	**Source**	**Identifier**	**Sequence (5' to 3')**
shTGFBI-417	Sigma-Aldrich	TRCN0000291417	CCGGGCGCTTGAGATCTTCAAACAACTCG<br>AGTTGTTTGAAGATCTCAAGCGCTTTTTG
shTGFBI-419	Sigma-Aldrich	TRCN0000291419	CCGGAGAAGGTTATTGGCACTAATACTCG<br>AGTATTAGTGCCAATAACCTTCTTTTTG
shITGAV-1	Sigma-Aldrich	TRCN0000010768	CCGGGTGAGGTCGAAACAGGATAAACTC<br>GAGTTTATCCTGTTTCGACCTCACTTTTT
shITGAV-3	Sigma-Aldrich	TRCN0000010769	CCGGCGACAGGCTCACATTCTACTTCTCG<br>AGAAGTAGAATGTGAGCCTGTCGTTTTT

Table S6 The list of primer sequences used for the qRT-PCR.

Gene	Forward primer (5' to 3')	Reverse primer (5' to 3')
TGFBI	ATGACCCTCACCTCTATGTACC	CACAGTTCACAGTTACAATCCCA
CD163	CATGTCTCTGAGGCTGACCA	TGCACACGATCTACCCACAT
CD206	TACCTGAGCCCACACCTGCT	GCGCGTTGTCCATGGTTTCC
CD86	CCCGGATGGTGTGTGGCATA	TCACAAGGAGGAGGGCCACA
iNOS	TTCAGTATCACAACCTCAGCAAG	TGGACCTGCAAGTTAAAATCCC
SOX2	TACAGCATGTCCTACTCGCAG	GAGGAAGAGGTAACCACAGGG
GFAP	CTGCGGCTCGATCAACTCA	TCCAGCGACTCAATCTTCCTC
ITGAV	GCTGTCGGAGATTTCAATGGT	TCTGCTCGCCAGTAAAATTGT
ITGA3	CTACCACAACGAGATGTGCAA	CCGAAGTACACAGTGTTCTGG
ITGA5	GGCTTCAACTTAGACGCGGAG	TGGCTGGTATTAGCCTTGGGT
ITGA6	GGCGGTGTTATGTCCTGAGTC	AATCGCCCATCACAAAAGCTC
ITGB1	GTGTGGAAGTGGTCTACCTGG	CAGCAGCGAGTTCTGAATGTC
ITGB3	AATCTCCTGTGCATCACATTTCT	TTCATAGCTGTCAAGATGACGTG
ITGB5	TCTCGGTGTGATCTGAGGG	TGGCGAACCTGTAGCTGGA
ACTB	CTCCTCCGAGTCAACAGATTCA	CAACAGCTTCTGAGGTAGGGA
GAPDH	AAGGTGAAGGTCGGAGTCAAC	GGGGTCATTGATGGCAACAATA

References

[1]. Pyonteck SM, Akkari L, Schuhmacher AJ, *et al.* CSF-1R inhibition alters macrophage polarization and blocks glioma progression. *Nat Med*. 2013 **19:** 1264-1272.

[2]. Darmanis S, Sloan SA, Croote D, *et al.* Single-Cell RNA-Seq Analysis of Infiltrating Neoplastic Cells at the Migrating Front of Human Glioblastoma. *Cell reports*. 2017 **21:** 1399-1410.
